# Validation of the NovaSeq6000 platform and automated library preparation for CE-IVD equivalence

**DOI:** 10.1016/j.csbj.2025.10.051

**Published:** 2025-11-01

**Authors:** Elena Pasquinelli, Giulia Rollo, Flavia Tinella, Miriana Danelli, Samantha Minetto, Giulia Casamassima, Michel Bader, Laura Calonaci, Olga Lorenza Colavecchio, Rossella Tita, Roberta Mancini, Margherita Baldassarri, Caterina Lo Rizzo, Anna Maria Pinto, Francesca Ariani, Chiara Fallerini, Paola Montagna, Vincenzo Mezzatesta, Mirella Bruttini, Alessandra Renieri

**Affiliations:** a Medical Genetics, University of Siena, Siena, Italy; b Med Biotech Hub and Competence Centre, Department of Medical Biotechnologies, University of Siena, Siena, Italy; c Genetica Medica, Azienda Ospedaliera Universitaria Senese, Siena, Italy; d Dipartimento Medicina Molecolare e dello Sviluppo, Università di Siena, Italy; e Independent Senior Consultant, Freelance Professional, Italy; f Direzione Generale, Azienda Ospedaliera Universitaria Senese, Siena, Italy

**Keywords:** Next-generation sequencing, NovaSeq6000, NovaSeq6000Dx, Whole-exome sequencing

## Abstract

The implementation of next-generation sequencing (NGS) technologies in clinical diagnostics requires rigorous validation of sequencing platforms and analytical workflows. In this study, we validated the performance of the Illumina NovaSeq6000 Research Use Only (RUO) platform, combined with automated library preparation using the Hamilton Microlab STAR system, by comparison to the CE-IVD certified NovaSeq6000Dx platform, which currently relies on manual library preparation. A total of 96 clinical samples underwent whole-exome sequencing (WES) on both platforms. Variant detection performance was assessed for single nucleotide variants (SNVs) and copy number variants (CNVs). The RUO platform demonstrated 100 % concordance with the CE-IVD system for clinically relevant SNVs, with full agreement across positive, negative, and overall percent agreement metrics. For CNVs larger than 150 kb, the positive percent agreement was 79 %, rising to 91.7 % for CNVs > 900 kb. Coverage uniformity and autosomal callability were consistently high across platforms. These results confirm the analytical equivalence of the NovaSeq6000 RUO configuration with automated library preparation for clinical-grade WES. This validation framework supports the adoption of scalable, cost-effective workflows that can achieve diagnostic performance comparable to CE-IVD certified systems and may facilitate routine implementation of exome sequencing in clinical laboratories.

## Graphical Abstract

**Figure:**
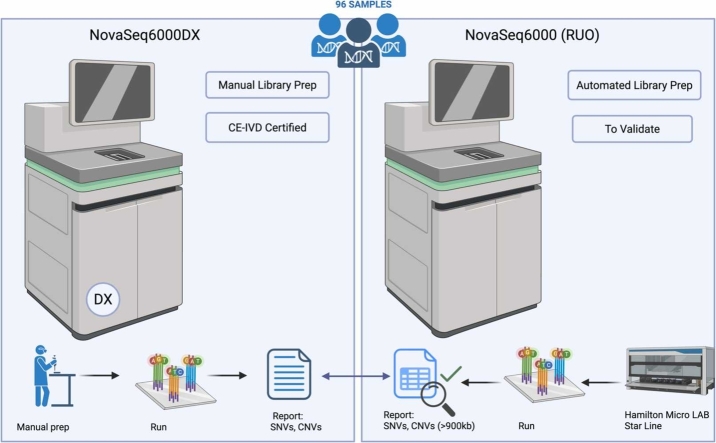

## 1 Introduction

The advent of Next-Generation Sequencing (NGS) technologies has revolutionized the field of clinical genetics, enabling broad-scale genomic investigations that significantly improve the diagnostic yield for patients with suspected genetic disorders [1]. Among the most widely adopted NGS applications in clinical practice is whole exome sequencing (WES), which targets the protein-coding regions of the genome and has proven to be a powerful tool for identifying pathogenic variants in a wide range of Mendelian conditions [2]. However, the implementation of WES for diagnostic purposes requires rigorous validation of both the sequencing platform and the associated laboratory workflows, to ensure compliance with regulatory standards and to guarantee the reliability, accuracy, and reproducibility of results [3].

This procedure arises from the need to internally validate the exome-based diagnostic process established at the Medical Genetics Unit (U.O.C. Genetica Medica) of the Azienda Ospedaliera Universitaria Senese (AOUS) Italy, with particular attention to the use of an internally available sequencing platform that is not CE-IVD marked. Specifically, the NovaSeq6000 Sequencing System by Illumina, does not possess the CE-IVD certification required for in vitro diagnostic use in Europe. Consequently, in light of the recent release of the NovaSeq6000Dx System, an updated CE-IVD certified version of the same platform, it is essential to undertake a comprehensive internal validation process to evaluate the performance characteristics and diagnostic suitability of the existing non-CE-IVD system [4], [5].

The validation process also encompasses the automated library preparation system, Hamilton Microlab Star, which plays a critical role in sample processing and contributes to the overall quality and reproducibility of sequencing results [6]. The goal of this technical procedure is to establish standardized operational guidelines for performing a comparative evaluation, risk assessment, and functional verification protocol. This will support the internal validation of the NovaSeq6000 system relative to the NovaSeq Dx model released by Illumina in 2022.

According to the European In Vitro Diagnostic Regulation (IVDR 2017/746), CE-IVD certification applies to the entire diagnostic workflow, including the sequencing instrument, reagents, and library preparation process. While Illumina has declared the NovaSeq6000 RUO and NovaSeq6000Dx to be technically equivalent at the hardware level, the associated workflows differ: the CE-IVD system requires manual library preparation with certified reagents, whereas our internal pipeline combines the RUO instrument with automated library preparation. Because certification cannot be extended to mixed configurations, an internal validation against the CE-IVD reference workflow was necessary to provide documented evidence of analytical equivalence and to ensure regulatory compliance.

This internal validation initiative is crucial for maintaining high diagnostic standards within the AOUS Medical Genetics Laboratory and for ensuring the continued reliability of genetic test results provided to clinicians and patients. In addition, in our laboratory the pre-analytical phase is also automated: genomic DNA is routinely extracted from peripheral blood using the MagCore® automated nucleic acid extraction system (Diatech Pharmacogenetics, Jesi, Italy). This ensures standardized, reproducible, and quality-controlled DNA input for downstream whole-exome sequencing, further supporting the establishment of a fully automated end-to-end workflow. The publication of this validation framework may provide valuable guidance to the broader scientific and medical community by offering a reproducible and transparent model for validating WES workflows in clinical settings.

As the use of WES becomes increasingly widespread in diagnostic practice, sharing structured validation approaches contributes to the harmonization of procedures and promotes quality assurance across laboratories.

This internal process was designed in alignment with the principles of the EU In Vitro Diagnostic Regulation (IVDR 2017/746), particularly Annex I (General Safety and Performance Requirements) and Annex XIII (Performance Evaluation) [5]. Although this study does not represent a formal CE-IVD certification procedure, the validation framework was structured to address analytical performance and clinical performance, thereby ensuring consistency with the regulatory expectations for diagnostic workflows.

Here, we hypothesize that the Illumina NovaSeq6000 RUO platform, combined with fully automated library preparation using the Hamilton Microlab STAR system, provides analytical performance equivalent to the CE-IVD certified NovaSeq6000Dx workflow based on manual library preparation. The primary objective is to validate the internal semi-automated WES workflow at AOUS by assessing concordance with a CE-IVD reference workflow for single nucleotide variants (SNVs) and copy number variants (CNVs). The secondary objectives are: evaluating sequencing run quality metrics and coverage uniformity; quantifying performance across different CNV size thresholds (>150 kb and >900 kb); assessing the diagnostic yield compared to published benchmarks.

For the present internal project, we established explicit parameters to guide the validation of the NovaSeq6000 workflow: 1) Analytical accuracy: demonstration of concordance in variant detection (SNVs and CNVs) with a CE-IVD certified reference system. 2) Sensitivity and specificity thresholds adequate performance for SNVs (>99 % concordance) and CNVs within accepted ranges for WES-based pipelines (typically 75–90 % depending on event size). 3) Coverage and callability metrics: evaluation of mean depth, uniformity of coverage, duplication rates, and proportion of callable bases in autosomal regions. 4) Clinical performance: demonstration that diagnostic yield is consistent with published benchmarks for exome sequencing (≈30–40 %). These parameters were defined as part of the institutional validation plan and are consistent with criteria adopted in previous validation studies of NGS workflows reported in the literature [7], [8].

## 2 Materials and methods

### 2.1 Study design

**Fig. 1:**
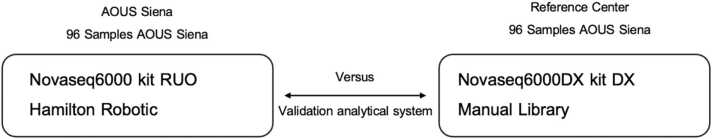
Comparative workflow for analytical validation using 96 AOUS Siena samples. The automated pipeline (left) utilizes the NovaSeq6000 RUO kit with Hamilton robotic library preparation, while the reference pipeline (right) uses the NovaSeq6000DX DX kit with manual library preparation.

This validation protocol aims to evaluate the analytical performance of the NovaSeq6000 Sequencing System (Illumina), currently in use at the Medical Genetics Unit of the Azienda Ospedaliera Universitaria Senese (AOUS), by comparing its outputs with those generated by a CE-IVD marked NovaSeq6000Dx system, used in an accredited external Reference Center. The analysis focuses on whole-exome sequencing (WES) across 96 samples, reflecting routine clinical diagnostic scenarios (Fig. 1).

### 2.2 Sample selection and composition

A total of 96 samples were selected to reflect realistic diagnostic scenarios encountered in clinical practice. The cohort included both individual and family-based cases. Specifically, 51 unrelated individuals were analyzed, including patients with cancer, alongside 15 family units composed of trios or quads, primarily related to neurodevelopmental disorders. In these cases, the probands were analyzed in conjunction with parental samples, following a TRIO-based study design. All 96 samples originated from the AOUS Medical Genetics Laboratory at AOUS and were processed in parallel on both sequencing platforms using aliquots of the same genomic DNA. This approach ensured that technical variability between runs was minimized, and that observed differences in variant detection were attributable solely to platform-specific performance and not to pre-analytical differences in sample preparation.

### 2.3 DNA extraction

Genomic DNA was extracted from peripheral blood samples using the MagCore® automated nucleic acid extraction system (Diatech Pharmacogenetics, Jesi, Italy), following the manufacturer’s standard protocol for whole blood. The MagCore platform provides fully automated extraction with magnetic-bead based purification, ensuring high reproducibility and minimal operator handling. Extracted DNA was quantified using the Qubit™ dsDNA HS Assay Kit (Thermo Fisher Scientific, Waltham, MA, USA).

### 2.4 Sequencing platforms

The platform under validation was the NovaSeq6000 Sequencing System, located at the Medical Genetics Unit of AOUS Siena (Table 1). Although not CE-IVD certified, Illumina has declared that this instrument is technically equivalent in hardware and performance to the NovaSeq6000Dx, the reference platform used for comparison. The NovaSeq6000Dx system is CE-IVD marked and installed in an accredited public or private diagnostic laboratory, serving as the gold standard for validation.

**Table 1:** Whole exome sequencing workflow under validation at AOUS siena.

**Pre-Analytical Phases**			
**Nr.**	**Step**	**Description**	**Automation (Hamilton STAR)**
1	Sample selection	Samples for one experiment	No
2	Hamilton robotic setup	Hamilton Microlab STAR User Manual	Yes
3	DNA-index association	1000000048041 v07 Illumina Manual	Yes
4	Indexed sample concentration measurement	Qubit 1X dsDNA HS Assay Kit	No
5	Dilution	1000000048041 v07 Illumina Manual	Yes
6	Pool preparation	1000000048041 v07 Illumina Manual	Yes
7	Purification	1000000048041 v07 Illumina Manual	Yes
8	PCR enrichment	1000000048041 v07 Illumina Manual	Yes
9	Purification	1000000048041 v07 Illumina Manual	Yes
10	Quantification and Bioanalyzer analysis	1000000048041 v07 Illumina Manual	No
11	Dilution and Pooling	1000000048041 v07 Illumina Manual	Yes
12	Quantification and Bioanalyzer analysis	1000000048041 v07 Illumina Manual	No
13	Final dilution	1000000048041 v07 Illumina Manual	No
**Analytical Phases**			
1	Sample-Sheet Preparation	1000000106351 v04 Illumina Manual	No
2	Reagent thawing	1000000019358 v.16 Illumina Manual	No
3	Novaseq6000 setup	1000000019358 v.16 Illumina Manual	No
4	Pool loading	1000000019358 v.16 Illumina Manual	No
5	Final check and run initiation	1000000019358 v.16 Illumina Manual	No
**Post-Analytical Phases**			
1	Primary analysis (BCL to FASTq)	GenomeUp JuliaOmix	No
2	Secondary analysis (BAM/CRAM files, VCF files)	GenomeUp JuliaOmix	No
3	Tertiary analysis (Annotation, filtering, classification)	GenomeUp JuliaOmix	No

**Notes**: This table summarizes the complete WES workflow under internal validation at AOUS Siena, structured into pre-analytical, analytical, and post-analytical phases. Laboratory steps follow Illumina and Hamilton protocols, while all bioinformatic analyses are performed using the GenomeUp JuliaOmix platform.

At AOUS, sequencing libraries were prepared using the Illumina DNA Prep with Exome 2.5 Enrichment Kit (RUO), Set B for 96 samples (Cat. No. 20077595), combined with the Twist Bioscience Exome 2.5 Panel (Cat. No. 20076914) for target enrichment. Sequencing was performed on the NovaSeq 6000 S2 Reagent Kit v1.5 (300 cycles), RUO (Cat. No. 20028314). Library preparation steps were fully automated using the Hamilton Microlab STAR system (NGS STAR model).

In contrast, the Reference Center employed a manual library preparation workflow using the Illumina DNA Prep with Enrichment Dx with UD Indexes Set A (96 samples, Cat. No. 20051352), paired with the same Twist Exome 2.5 Panel. Sequencing was carried out using the NovaSeq 6000Dx S2 Reagent Kit v1.5 (300 cycles), CE-IVD certified (Cat. No. 20046931).

### 2.5 Bioinformatic analysis

Sequencing data generated from both AOUS Siena and the Reference Center underwent standardized bioinformatic using the JuliaOmix platform developed by GenomeUp processing to ensure consistency and minimize technical bias. Primary analysis, including base calling and conversion of BCL to FASTQ files, was performed using the Illumina DRAGEN platform (v4.3.6), which also supported secondary analysis tasks such as alignment to the reference genome (GRCh37), duplicate marking, and variant calling [9]. For comparison and refinement, variant calling was further validated using GATK v4.4 and DeepVariant v1.5, enabling robust detection of single nucleotide variants (SNVs) and small insertions/deletions (indels) [10], [11]. These variant sets were harmonized and annotated using Ensembl Variant Effect Predictor (VEP) v113, which provided functional consequences for each variant based on the GRCh37 reference [12].

For downstream analysis, only variants concordantly identified by at least two out of three pipelines (DRAGEN, GATK, DeepVariant) were retained, in order to minimize caller-specific artifacts and ensure high-confidence calls.

Comprehensive variant annotation included integration of multiple population and clinical databases. Population allele frequencies were retrieved from both gnomAD v2.1.1 (GRCh37) and v4.1 (GRCh37), while known variant references were queried against dbSNP build 146 and ClinVar (release 20250209) [13], [14], [15]. Predictive functional scores for missense and splice-altering variants were incorporated from dbNSFP v4.4 and dbscSNV v1.1, supporting variant prioritization and clinical interpretation. Only variants classified as ACMG classes 3, 4, or 5 and relevant to the clinical indication of each patient were retained for comparative analysis. These represent clinically significant findings that would be considered reportable in a real-world diagnostic setting. For each such variant, zygosity, functional annotation, and key quality metrics were documented and summarized in Supplementary Table 1.

Copy number variant (CNV) detection was performed using the Illumina DRAGEN platform (v4.3.6) applied to whole-exome sequencing (WES) data generated with the Illumina Exome 2.5 Panel (GRCh37). CNV segmentation was carried out using the high-sensitivity low-mappability (HSML) mode, with the segmentation window size set to 500 base pairs (-cnv-width-size 500). This setting divided the total target regions (∼262 Mb) into approximately 525,210 windows, enabling genome-wide assessment of copy number profiles at moderate resolution. CNV merging was disabled by default, in accordance with Illumina recommendations for WES analysis, given the spacing between targeted intervals. The merge threshold was maintained at the default linear copy ratio difference of 0.4, allowing for detection of focal CNVs while reducing the risk of artificial fusion of distant segments. For validation purposes, only CNVs classified as clinically relevant and associated with the patient’s diagnostic question were considered. These events would be deemed reportable in a clinical setting, and include deletions or duplications ≥ 150 kb overlapping known disease-associated loci and are reported in Supplementary Table 2.

### 2.6 Analytical system validation declaration

This validation was qualitative in nature and focused on the presence or absence of expected variants, as determined by a reference table containing 96 pseudonymized samples and their known variant profiles. Sequencing results obtained at AOUS were directly compared to those generated using the CE-IVD certified NovaSeq6000Dx system at an external accredited Reference Center, following Illumina’s standard validation protocol. The assessment of analytical performance included evaluation of accuracy, precision and clinical performance [16]. Accuracy was determined by the concordance of variant calls between the two systems. Precision was measured by the percentage of positive calls (PPC), defined as the number of correctly identified variants over the total number of evaluable variant positions. Clinical performance indicators were also calculated. Positive Percent Agreement (PPA) was defined as the proportion of variant loci correctly reported by the test system. Negative Percent Agreement (NPA) referred to the correct identification of wild-type loci. Overall Percent Agreement (OPA) represented the combined concordance for both variant and wild-type loci. Additionally, the percentage of positive and negative calls (PPC and PNC), excluding low-confidence or low-depth regions, was used to further quantify analytical robustness. The callability of autosomal regions was also evaluated as the proportion of non-N bases in targeted autosomal intervals with valid genotype calls.

## 3 Results

### 3.1 Run metrics

Run metrics from the two sequencing workflows demonstrated overall comparability in terms of read alignment and depth of coverage. The total number of aligned reads was similar between the two runs, while the average alignment depth and mean coverage across autosomal and sex chromosomes were higher in the AOUS Siena dataset. The percentage of target bases achieving standard depth thresholds (≥15 ×, ≥20 ×, ≥50 ×, and ≥100 ×) remained consistently high in both datasets, with minimal variation observed across platforms. A slight increase in duplication rate was noted in the AOUS Siena run (Table 2).

**Table 2:** Run metrics comparison between the AOUS automated workflow and the CE-IVD manual workflow.

Metrics	REF Center	AOUS Siena
Aligned Reads	79243944.61	76562561.09
Mean Alignment Depth	124.75	139.65
Autosomal Mean Coverage	125.94	140.89
Chr X Mean Coverage	100.87	115.09
Chr Y Mean Coverage	14.89	17.34
Chr M Mean Coverage	0.0	0.0
Number of Marked Duplicate Reads	8503316.64	14338459.55
Percentage of marked duplicate reads	9.01	14.92
Percentage of Bases in target_bed with ≥ 15 × Coverage	98.19	97.35
Percentage of Bases in target_bed with ≥ 20 × Coverage	97.88	96.58
Percentage of Bases in target_bed with ≥ 50 × Coverage	91.11	86.39
Percentage of Bases in target_bed with ≥ 100 × Coverage	60.27	60.49

Note: Values represent sequencing run-level statistics obtained from one 96-sample experiment per workflow, as reported by the Illumina sequencing output. Metrics include aligned reads, mean alignment depth, coverage across autosomal and sex chromosomes, duplication rate, and coverage at standard thresholds (≥15 ×, ≥20 ×, ≥50 ×, ≥100 ×).

### 3.2 SNV validation

To assess the analytical validity of single nucleotide variant (SNV) detection, we compared the results obtained from the NovaSeq6000 system at AOUS Siena with those from the CE-IVD certified NovaSeq6000Dx platform used in the Reference Center. A complete list of detected SNVs for each sample, including genotype concordance, is provided in Supplementary Table 1. Out of 35 clinically relevant samples, all variants identified at the Reference Center were also correctly detected by the AOUS platform. This resulted in a Positive Percent Agreement (PPA) of 100.00 %.

Among 61 samples classified as negative by the Reference Center, all were correctly confirmed as negative by the AOUS system, yielding a Negative Percent Agreement (NPA) of 100.00 %.

**Fig. 2:**
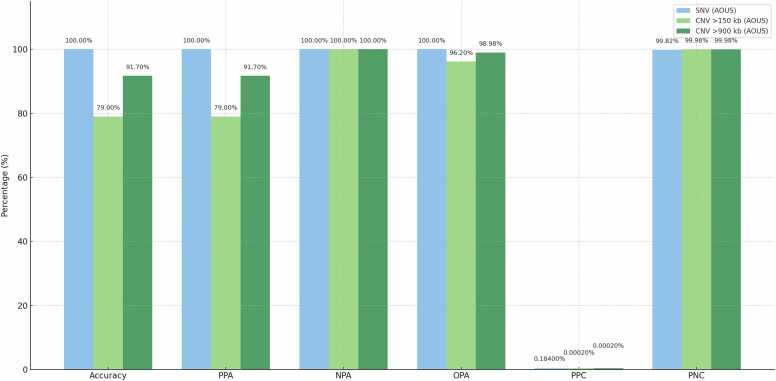
Comparative performance metrics for SNV and CNV detection.

Combining these results, the Overall Percent Agreement (OPA) including both positive and negative concordant calls was 100.00 % (Fig. 2).

Analytical performance metrics calculated over all tested base positions (n = 36,547,587) showed a Percent Positive Calls (PPC) of 0.181 % and a Percent Negative Calls (PNC) of 99.8187 % for the AOUS platform. These values were comparable to those obtained on the reference NovaSeq6000Dx system with a consistent distribution of variant and wild-type calls between the two workflows. The callability of autosomal regions at ≥ 20 × coverage was 96.58 %**,** slightly below the 97.88 % obtained on the reference platform.

### 3.3 CNV Validation (>150 kb)

As part of the extended analytical validation, copy number variant (CNV) detection was evaluated for events larger than 150 kb. A total of 96 samples were evaluated for copy number variant (CNV) detection on both the NovaSeq6000Dx (Reference Center) and the NovaSeq6000 RUO platform (AOUS).

The reference center identified 19 clinically relevant CNVs, of which 15 were correctly detected by AOUS, resulting in an accuracy and Positive Percent Agreement (PPA) of 79 %.

The AOUS system classified 88 samples as negative, but only 86 of these were true negatives, indicating two false negative results. Full CNV call data, including discordant events, are shown in Supplementary Table 2. The resulting Negative Percent Agreement (NPA) was 100 %, calculated based on the 86 confirmed negatives over 86 expected negatives. The Overall Percent Agreement (OPA) across the entire cohort was 96.20 % (Fig. 2). The Percent Positive Calls (PPC) and Percent Negative Calls (PNC) were calculated globally across all CNV calls on the AOUS platform, based on 525,210 genomic segments. PPC was 0.0002 % and PNC was 99.98 %. The callability of autosomal regions at ≥ 20 × coverage was 96.58 % for AOUS, compared to 97.88 % on the reference platform.

### 3.4 CNV Validation (>900 kb)

Among the 96 samples analyzed, the reference center identified 12 CNVs larger than 900 kb, of which 11 were correctly detected by the AOUS platform. This corresponds to a Positive Percent Agreement (PPA) and analytical accuracy of 91.7 %. The AOUS system classified 93 samples as negative, all of which were also negative according to the reference platform, yielding a Negative Percent Agreement (NPA) of 100 % for CNVs larger than 900 kb. One CNV > 900 kb was not identified by the AOUS workflow, resulting in a false negative. The Overall Percent Agreement (OPA) between the two systems for this subset was 98.98 % (Fig. 2). As noted above, PPC and PNC values (0.0002 % and 99.98 %, respectively) were calculated across all CNV calls, including those in this > 900 kb subset, based on the 525,210 genomic segments evaluated. The callability of autosomal regions at ≥ 20 × coverage remained at 96.58 % for the AOUS platform, versus 97.88 % on the reference system.

Bar chart comparing key validation parameters (Accuracy, PPA, NPA, OPA, PPC, PNC) across three variant types (SNVs, CNVs >150 kb, CNVs >900 kb). All values represent concordance of the AOUS automated RUO workflow against the CE-IVD manual workflow, which was used as the reference standard (gold standard). Thus, CE-IVD results are inherently represented as baseline values in each metric. While all categories reached 100 % NPA, a gradual improvement in PPA and OPA is observed from CNVs > 150 kb to CNVs > 900 kb, reflecting improved detectability with increasing variant size. SNV performance was uniformly optimal across all metrics.

### 3.5 Diagnostic yield analysis

To evaluate the clinical performance of the assay across different referral indications, we calculated the diagnostic yield per clinical cohort.

A total of 74 probands were analyzed, with an overall diagnostic yield of 32.4 %.

Stratified by indication, yields were 50.0 % in dyslipidemias, 35.1 % in hereditary cancer, and 33.3 % in cardiomyopathies and neurodevelopmental disorders (Table 3). When stratified by variant type, all clinically relevant SNVs identified by the Reference Center were equally detected by the AOUS Siena workflow, confirming a 100 % diagnostic concordance between the two systems. Although minor differences in CNV detection accuracy were observed during analytical validation, none of the discordant CNV events contributed to clinical diagnoses.

**Table 3:** Diagnostic yield across all cohorts.

Clinical cohort	Total cases (N)	Positive diagnoses (N)	Diagnostic yield (%)
Hereditary cancer / oncologic predisposition	37	13	**35.1 %**
Cardiomyopathies / inherited arrhythmias	9	3	**33.3 %**
Neurodevelopmental disorders	9	3	**33.3 %**
Autoinflammatory / metabolic diseases	9	2	**22.2 %**
Dyslipidemias	4	2	**50.0 %**
Other conditions	6	1	**16.7 %**
Overall total	**74**	**24**	**32.4 %**

## 4 Discussion

This study demonstrates that the NovaSeq6000 platform, combined with an automated library preparation workflow, achieves excellent analytical performance that is largely equivalent to the CE-IVD certified NovaSeq6000Dx system.

This study focused on the analytical validation of SNVs and CNVs detectable through a WES-based pipeline. Other classes of structural variants (SVs), such as inversions, translocations, and complex insertions, were not assessed, as these typically require whole-genome sequencing or long-read technologies for accurate resolution.

For single nucleotide variants (SNVs), the Positive Percent Agreement, Negative Percent Agreement, and Overall Percent Agreement all reached 100 %, underscoring the capacity of the workflow to reliably identify clinically relevant variants. This is consistent with previously published studies reporting high concordance rates (>99 %) between RUO and CE-IVD configurations of Illumina platforms [17].

For copy number variants (CNVs) larger than 150 kb, the Positive Percent Agreement was 79 %, rising to 91.7 % for events exceeding 900 kb, and the Overall Percent Agreement approached 99 %. The observed Positive Percent Agreement (PPA) of 79 % for CNVs larger than 150 kb may appear moderate for clinical validation. However, this value aligns with expectations for CNV detection from whole-exome sequencing (WES) data, where sensitivity typically ranges from 75 % to 90 %, depending on event size, genomic context, and bioinformatic strategy [18], [19].

Prior studies have demonstrated that detection sensitivity increases significantly for CNVs ≥ 100 kb, as smaller events often fall below the resolution limits of exon-level read-depth analysis due to sparse target coverage and increased signal variability [20]. While the literature supports 100 kb as a general lower boundary, we adopted a more conservative threshold of 150 kb to minimize false negatives and prioritize events with greater diagnostic impact. This choice reflects a balance between analytical reliability and clinical utility.

Furthermore, sensitivity increased to 91.7 % for CNVs exceeding 900 kb, which are more robustly detected due to clearer coverage signals and better alignment performance. These findings underscore the appropriateness of our validation framework for capturing clinically reportable CNVs with meaningful diagnostic impact. These large events typically generate more robust and uniform read-depth signals, enhancing detection confidence in WES-based pipelines. We selected the 900 kb threshold based on array studies reporting 90–95 % sensitivity and specificity for CNVs ≥ 900 kb [21].

These results reflect the known limitations of exome-based CNV detection, which tends to perform optimally on large events with clear coverage shifts, while smaller or complex rearrangements remain more challenging to resolve accurately [22], [23].

Notably, among the 12 CNVs ≥ 900 kb identified by the reference center, ten were concentrated within a single sample, consistent with a chromothripsis-like event. In this case, the CE-IVD system detected ten distinct rearrangements, while the RUO configuration identified eight of them. Although two breakpoints were not confirmed by the RUO pipeline, the overall pattern of complex genomic rearrangement was consistently captured.

No additional orthogonal validation was performed on discordant CNV calls because the CE-IVD certified NovaSeq6000Dx platform served as the reference method. Given its regulatory approval and use in an accredited diagnostic center, the CE-IVD workflow was considered an appropriate benchmark for analytical comparison. This approach is consistent with prior studies that have used clinically validated platforms as reference comparators for assessing research-use-only (RUO) systems, particularly when aiming to assess equivalence rather than absolute performance [18], [19].

The overall diagnostic yield across 74 probands was 32.4 %, which falls within the expected range reported for heterogeneous clinical cohorts undergoing multigene or exome-based testing. Previous large-scale exome studies have reported diagnostic yields between 25 % and 40 %, depending on case selection, gene panel size, and inclusion of copy number variant analysis [24], [25].

When stratified by clinical indication, our results were broadly consistent with the literature, although some categories displayed higher yields due to cohort enrichment and clinical pre-selection. In hereditary cancer syndromes, the diagnostic yield reached 35.1 %, which is higher than that observed in population-based or pan-cancer cohorts, where rates typically range from 10 to 25 % [26], [27]. This difference is likely explained by the strict inclusion of patients with strong familial or clinical suspicion and by the use of a broad, comprehensive in silico gene panel encompassing mismatch-repair and homologous recombination repair pathways. For cardiomyopathies and inherited arrhythmias, we observed a yield of 33.3 %, which is fully in line with published data showing rates between 20 % and 40 %, depending on the predominant phenotype and gene coverage [28], [29]. The diagnostic yield for neurodevelopmental disorders was 33.3 %, which closely mirrors the results of meta-analyses and large sequencing cohorts reporting rates between 31 % and 36 % [30], [31]. These similarities support the robustness of the sequencing and variant interpretation workflow across diverse phenotypes, despite the smaller sample size in this subgroup. For autoinflammatory and metabolic disorders, we obtained a yield of 22.2 %, again consistent with the upper range of published values (15–23 %) for similar gene panels [32].

In familial dyslipidemias, the observed diagnostic yield of 50.0 % falls within the high end of published reports. Genetic confirmation rates in clinically suspected familial hypercholesterolemia vary from 28 % to 80 % depending on pre-test probability and clinical criteria [33], [34]. These comparisons support the conclusion that the validated pipeline not only demonstrates analytical equivalence but also maintains high diagnostic utility.

Evaluating run quality metrics, we observed that the AOUS platform had a higher duplication rate (14.9 %) compared to the CE-IVD system (9.0 %). This difference did not affect variant detection, as overall coverage depth, callability, and variant concordance remained fully comparable. Slightly elevated duplication rates are commonly observed in automated workflows, particularly when using robotic liquid handling platforms for library preparation. A similar finding was reported by Zuckerman et al., who observed duplication rates of 31 % with an automated capture-based workflow compared to 28 % with a manual protocol. Their study attributed this modest increase not to an intrinsic limitation of automation, but to physical and thermodynamic factors such as microenvironmental differences between a heated plate on a robotic system and a sealed thermal cycler, which may reduce library complexity and increase PCR duplicates [35]. Importantly, the duplication rate in our automated workflow remained well within accepted quality control thresholds for clinical WES, as Illumina Sequencing Coverage and Analysis Recommendations indicate that values below 20 % are considered acceptable for exome and genome sequencing [36].

One limitation of this study is the absence of intra-run or inter-run replicates, which are critical to formally assess reproducibility and precision over time and are often required by regulatory standards. In this initial validation, our primary objective was to demonstrate analytical equivalence with the CE-IVD NovaSeq6000Dx workflow, which guided the study design. Nonetheless, additional replicate experiments are planned in ongoing validation phases, with the aim of providing a more comprehensive evaluation of technical precision and reproducibility across runs.

A further limitation of this work concerns the absence of an explicit validation of small indels and mid-sized CNVs. Reliable detection of these variant classes with short-read WES remains technically challenging and requires specific bioinformatic optimization and orthogonal confirmation [37]. In our accredited diagnostic practice, clinically relevant small indels are systematically verified by orthogonal methods (e.g., Sanger sequencing), while CNVs < 150 kb are routinely assessed by complementary technologies such as array-CGH or MLPA. Moreover, the analytical validation in this study was restricted to causative variants associated with the clinical question of each case. As no causative delins variants were observed in our dataset, their inclusion in the validation analysis was not possible. While we acknowledge this limitation, our approach reflects current diagnostic workflows and ensures that clinically relevant findings are appropriately confirmed. A full analytical validation of indel detection, although of interest, was beyond the scope of the present project.

Another point is the absence of orthogonal confirmation for discordant CNV calls. In our design, the CE-IVD certified NovaSeq6000Dx workflow, implemented in an accredited diagnostic center, was adopted as the gold standard comparator. Given its regulatory approval and established use in routine diagnostics, this was considered an appropriate benchmark for evaluating the RUO configuration. Nonetheless, we acknowledge that the lack of orthogonal confirmation may reduce confidence in attributing discrepancies solely to platform differences. Future validation phases may incorporate targeted orthogonal testing (e.g., array-CGH, MLPA) to further characterize discordant CNV calls and strengthen analytical confidence.

Taken together, our findings indicate that a fully automated WES workflow based on the NovaSeq6000 can achieve analytical performance comparable to the CE-IVD system for the variant classes explicitly validated in this study. Compared to the CE-IVD system, this configuration offers substantial advantages in scalability and cost containment, as it allows high-throughput processing without requiring manual library preparation. Furthermore, the ability to deliver diagnostic performance comparable to CE-IVD workflows suggests that laboratories operating under accreditation standards may consider similar validation strategies to optimize resource utilization while maintaining regulatory compliance.

## 5 Conclusion

The NovaSeq6000 demonstrated excellent analytical performance in detecting clinically relevant SNVs and high reliability for large CNVs, supporting its suitability for diagnostic WES in clinical setting. This study validates the NovaSeq6000 with automated Hamilton-based library preparation, providing a fully automated, scalable workflow more suitable for high-throughput clinical use than the CE-IVD system, which still relies on manual preparation. Moreover, validating the NovaSeq6000 allows clinical laboratories to achieve diagnostic performance comparable to DX-certified platform without compromising diagnostic accuracy and saving costs. This validation framework provides a model for harmonizing WES workflows in routine clinical genetics.

## CRediT authorship contribution statement

**Roberta Mancini:** Supervision, Validation, Visualization. **Rossella Tita:** Investigation, Supervision, Validation, Visualization. **Margherita Baldassarri:** Resources, Validation, Visualization. **Laura Calonaci:** Formal analysis, Methodology. **Olga Lorenza Colavecchio:** Formal analysis, Methodology. **Francesca Ariani:** Investigation, Project administration, Supervision, Validation, Visualization. **Chiara Fallerini:** Investigation, Methodology, Supervision, Validation, Visualization. **Caterina Lo Rizzo:** Resources, Supervision, Visualization. **Anna Maria Pinto:** Resources, Supervision, Visualization. **Giulia Rollo:** Data curation, Formal analysis, Methodology. **Flavia Tinella:** Data curation, Formal analysis, Investigation, Methodology. **Elena Pasquinelli:** Conceptualization, Data curation, Formal analysis, Investigation, Methodology, Project administration, Software, Supervision, Validation, Visualization, Writing – original draft, Writing – review & editing. **Giulia Casamassima:** Formal analysis, Investigation, Methodology. **Michel Bader:** Formal analysis, Methodology. **Miriana Danelli:** Data curation, Formal analysis, Investigation, Methodology. **Samantha Minetto:** Formal analysis, Methodology, Software. **Mirella Bruttini:** Data curation, Supervision, Validation, Visualization. **Alessandra Renieri:** Conceptualization, Data curation, Formal analysis, Funding acquisition, Investigation, Supervision, Validation, Visualization, Writing – review & editing. **Paola Montagna:** Investigation, Methodology, Supervision, Visualization. **Vincenzo Mezzatesta:** Methodology, Supervision, Validation, Visualization.

## Ethics statement

The study was conducted in accordance with the ethical principles established by the Declaration of Helsinki. All participants provided written informed consent, and the procedures were approved by the institutional ethics committee (Regional Ethics Committee for Clinical Trials of the Tuscany Region – South-East Area).

## Declaration of Competing Interest

The authors declare that they have no known competing financial interests or personal relationships that could have appeared to influence the work reported in this paper.

## Data Availability

The raw sequencing data generated in this study cannot be publicly shared due to restrictions in participant informed consent, which did not permit deposition in open-access repositories. Data access may be considered upon reasonable request and pending approval by the relevant ethics committee, subject to GDPR and institutional policies. However, detailed variant-level data are provided in the Supplementary Information which includes the complete set of clinically significant findings assessed in this study and serve as benchmarking subsets to support transparency and reproducibility.
