# Supplementary material for: Validation of the NovaSeq6000 platform and automated library preparation for CE-IVD equivalence
Source: Comput Struct Biotechnol J. 2025 Nov 1;27:4838–45. doi: 10.1016/j.csbj.2025.10.051 (PMC12639258; doi:10.1016/j.csbj.2025.10.051)
Supplement: Supplementary file 1 — Supplementary material [file mmc1.pdf]

	Results in reference center					Results in test center					Concordance
Code	Reported Gene	c.DNA	Protein	ACMG Class	Zygosity	Reported Gene	c.DNA	Protein	ACMG Class	Zygosity	
**Single samples**											
1	SEC23B	c.325G>A	p.Glu109Lys	5	heterozygous	SEC23B	c.325G>A	p.Glu109Lys	5	heterozygous	1
2	negative					negative					1
3	MSH2	c.2519_2530del	p.Val840_Cys843del	4	heterozygous	MSH2	c.2519_2530del	p.Val840_Cys843del	4	heterozygous	1
4	negative					negative					1
5	GLA	c.335G>A	p.Arg112His	5	hemizygous	GLA	c.335G>A	p.Arg112His	5	hemizygous	1
6	BRIP1	c.2087C>T	p.Pro696Leu	3	heterozygous	BRIP1	c.2087C>T	p.Pro696Leu	3	heterozygous	1
7	ERCC2	c.1308-2A>G	/	5	heterozygous	ERCC2	c.1308-2A>G	/	5	heterozygous	1
8	MYBPC3	c.1102_1104del	p.Lys368del	3	heterozygous	MYBPC3	c.1102_1104del	p.Lys368del	3	heterozygous	1
9	negative					negative					1
10	FLNC	c.3932C>T	p.Thr1311Ile	3	heterozygous	FLNC	c.3932C>T	p.Thr1311Ile	3	heterozygous	1
11	negative					negative					1
12	negative					negative					1
13	ERCC3	c.325C>T	p.Arg109Ter	5	heterozygous	ERCC3	c.325C>T	p.Arg109Ter	5	heterozygous	1
14	MUTYH	c.1187G>A	p.Gly396Asp	5	heterozygous	MUTYH	c.1187G>A	p.Gly396Asp	5	heterozygous	1
15	MUTYH	c.1420C>T	Arg474Cys	3	heterozygous	MUTYH	c.1420C>T	p.Arg474Cys	3	heterozygous	1
16	MLH3	c.1119_1122del	p.Phe374MetfsTer8	4	heterozygous	MLH3	c.1119_1122del	p.Phe374MetfsTer8	4	heterozygous	1
17	HOXB13	c.251G>A	p.Gly84Glu	3	heterozygous	HOXB13	c.251G>A	p.Gly84Glu	3	heterozygous	1
18	negative					negative					1
19	negative					negative					1
20	negative					negative					1
21	negative					negative					1
22	negative					negative					1
23	negative					negative					1
24	negative					negative					1
25	negative					negative					1
26	negative					negative					1
27	negative					negative					1
28	MUTYH	c.312C>A	p.Tyr104Ter	5	heterozygous	MUTYH	c.312C>A	p.Tyr104Ter	5	heterozygous	1
29	negative					negative					1
30	ongoing					negative					1
31	negative					negative					1
32	negative					negative					1
33	negative					negative					1
34	FANCF	c.224del	p.Gly75ValfsTer6	4	heterozygous	FANCF	c.224del	p.Gly75ValfsTer6	4	heterozygous	1
35	negative					negative					1
36	negative					negative					1
37	negative					negative					1
38	negative					negative					1
39	negative					negative					1
40	negative					negative					1
41	negative					negative					1
42	negative					negative					1
43	MSH6	c.3557G>A	p.Gly1186Asp	4	heterozygous	MSH6	c.3557G>A	p.Gly1186Asp	4	heterozygous	1
44	negative					negative					1
45	PTCH1	c.3114C>G	p.Cys1038Trp	3	heterozygous	PTCH1	c.3114C>G	p.Cys1038Trp	3	heterozygous	1
46	CHEK2	c.1180G>A	p.Glu394Lys	3	heterozygous	CHEK2	c.1180G>A	p.Glu394Lys	3	heterozygous	1
47	negative					negative					1
48	MAP2K1	c.803C>G	p.Ala268Gly	3	heterozygous	MAP2K1	c.803C>G	p.Ala268Gly	3	heterozygous	1
49	negative					negative					1
50	ATM	c.8581A>G	p.Ile2861Val	3	heterozygous	ATM	c.8581A>G	p.Ile2861Val	3	heterozygous	1
51	ERCC5	c.2751dup	p.Leu918IlefsTer12	4	heterozygous	ERCC5	c.2751dup	p.Leu918IlefsTer12	4	heterozygous	1
52	negative					negative					1
53	LDLR	c.1358+2T>C	/	4	heterozygous	LDLR	c.1358+2T>C	/	4	heterozygous	1
54	negative					negative					1
55	negative					negative					1
56	negative					negative					1
57	RAD51	c.776A>C	p.Glu259Ala	4	heterozygous	RAD51	c.776A>C	p.Glu259Ala	4	heterozygous	1
**Trios**											
58	LRP1<br>LRP1	c.6477C>A<br>c.10824C>A	p.Cys2159Ter<br>p.Asp3608Glu	4<br>3	heterozygous	LRP1<br>LRP1	c.6477C>A<br>c.10824C>A	p.Cys2159Ter<br>p.Asp3608Glu	4<br>3	heterozygous	1
59	LRP1	c.10824C>A	p.Asp3608Glu	3	heterozygous	LRP1	c.10824C>A	p.Asp3608Glu	3	heterozygous	1
60	LRP1	c.6477C>A	p.Cys2159Ter	4	heterozygous	LRP1	c.6477C>A	p.Cys2159Ter	4	heterozygous	1
61	negative					negative					1
62	negative					negative					1
63	negative					negative					1
64	negative					negative					1
65	negative					negative					1
66	GLA	c.335G>A	p.Arg112His	5	heterozygous	GLA	c.335G>A	p.Arg112His	5	heterozygous	1
67	NF1	c.3461A>G	p.Asn1154Ser	3	heterozygous	NF1	c.3461A>G	p.Asn1154Ser	3	heterozygous	1
68	NF1	c.3461A>G	p.Asn1154Ser	3	heterozygous	NF1	c.3461A>G	p.Asn1154Ser	3	heterozygous	1
69	negative					negative					1
70	TECR<br>TECR	c.66+25T>C<br>c.16-45C>G	/	3<br>3	heterozygous	TECR<br>TECR	c.66+25T>C<br>c.16-45C>G	/	3<br>3	heterozygous	1
71	TECR	c.66+25T>C	/	3	heterozygous	TECR	c.66+25T>C	/	3	heterozygous	1
72	TECR	c.16-45C>G	/	3	heterozygous	TECR	c.16-45C>G	/	3	heterozygous	1
73	negative					negative					1
74	negative					negative					1
75	negative					negative					1
76	negative					negative					1
77	negative					negative					1
78	negative					negative					1
79	MEFV	c.2080A>G	p.Met694Val	5	heterozygous	MEFV	c.2080A>G	p.Met694Val	5	heterozygous	1
80	negative					negative					1
81	MEFV	c.2080A>G	p.Met694Val	5	heterozygous	MEFV	c.2080A>G	p.Met694Val	5	heterozygous	1
82	LDLR	c.1567G>A	p.Val523Met	5	heterozygous	LDLR	c.1567G>A	p.Val523Met	5	heterozygous	1
83	negative					negative					1
84	LDLR	c.1567G>A	p.Val523Met	5	heterozygous	LDLR	c.1567G>A	p.Val523Met	5	heterozygous	1
85	negative					negative					1
86	negative					negative					1
87	negative					negative					1
**Quartet**											
88	negative					negative					1
89	negative					negative					1
90	negative					negative					1
91	negative					negative					1
**Quintet**											
92	negative					negative					1
93	negative					negative					1
94	negative					negative					1
95	negative					negative					1
96	negative					negative					1
