# Supplementary material for: Validation of the NovaSeq6000 platform and automated library preparation for CE-IVD equivalence
Source: Comput Struct Biotechnol J. 2025 Nov 1;27:4838–45. doi: 10.1016/j.csbj.2025.10.051 (PMC12639258; doi:10.1016/j.csbj.2025.10.051)
Supplement: Supplementary file 2 — Supplementary material [file mmc2.pdf]

Code Patient	Results in reference center (Rome)						Results in test center (Siena)						Concordance
	Location	Size	Disease gene(s) involved	ACMG Class	DEL/DUP	Genotype	Location	Size	Disease gene(s) involved	ACMG Class	DEL/DUP	Genotype	
**Single samples**													
1	Chr22:22988781:23657664	668883 (668 Kb)	BCR	5	DEL	Heterozygous	Chr22:22988781:23657664	668883 (668 Kb)	BCR	5	DEL	Heterozygous	1
2	chr8:42862989:43218462	355473 (355 Kb)	POMK, HGSNAT	5	DUP	Heterozygous	chr8:42862989:43218462	355473 (355 Kb)	POMK, HGSNAT	5	DUP	Heterozygous	1
3	negative						negative						1
4	negative						negative						1
5	negative						negative						1
6	DUP in chr 1, 2, 3, 4, 5, 6, 7, 8, 13, X	*	*	*	*	*	DUP in chr 2, 3, 4, 5, 6, 8, 13, X	*	*	*	*	*	1
7	chr8:42862989:43057679	194690 (194 Kb)	POMK, HGSNAT	5	DUP	Heterozygous	chr8:42862989:43218462	355473 (355 Kb)	POMK, HGSNAT	5	DUP	Heterozygous	1
8	negative						negative						1
9	chr8:42862989:43057679	194690 (194 Kb)	POMK, HGSNAT	5	DUP	Heterozygous	chr8:42862989:43218462	355473 (355 Kb)	POMK, HGSNAT	5	DUP	Heterozygous	1
10	negative						negative						1
11	negative						negative						1
12	negative						negative						1
13	negative						negative						1
14	negative						negative						1
15	negative						negative						1
16	negative						negative						1
17	Chr16:17564290:18426519	862229 (862 Kb)	XYLT1	5	DEL	Heterozygous	negative						0
18	negative						negative						1
19	negative						negative						1
20	negative						negative						1
21	negative						negative						1
22	negative						negative						1
23	negative						negative						1
24	negative						negative						1
25	chr17:18933743:19125677	191934 (191 Kb)	GRAP	4	DEL	Heterozygous	negative						0
26	negative						negative						1
27	negative						negative						1
28	negative						negative						1
29	negative						negative						1
30	chr5:69372845:70242003	869158 (869 Kb)	SMN1, SMN2	5	DEL	Heterozygous	chr5:69372845:70242003	869158 (869 Kb)	SMN1, SMN2	5	DEL	Heterozygous	1
31	negative						negative						1
32	chr2:41607:1442630	1401023 (1.4 Mb)	TPO	4	DEL	Heterozygous	chr2:41607:1442630	1401023 (1.4 Mb)	TPO	4	DEL	Heterozygous	1
33	negative						negative						1
34	negative						negative						1
35	negative						negative						1
36	negative						negative						1
37	negative						negative						1
38	negative						negative						1
39	negative						negative						1
40	negative						negative						1
41	negative						negative						1
42	negative						negative						1
43	negative						negative						1
44	negative						negative						1
45	negative						negative						1
46	negative						negative						1
47	negative						negative						1
48	negative						negative						1
49	negative						negative						1
50	negative						negative						1
51	negative						negative						1
52	negative						negative						1
53	ChrY:21205048:27770674	6565626 (6.5 Mb)	none	5	DEL	hemizygous	ChrY:21205048:27770674	6565626 (6.5 Mb)	none	5	DEL	hemizygous	1
54	negative						negative						1
55	negative						negative						1
56	negative						negative						1
57	negative												1
**Trios**													
58	negative						negative						
59	negative						negative						1
60	negative						negative						1
61	negative						negative						1
62	negative						negative						1
63	negative						negative						1
64	negative						negative						1
65	negative						negative						1
66	negative						negative						1
67	negative						negative						1
68	negative						negative						1
69	negative						negative						1
70	negative						negative						1
71	negative						negative						1
72	negative						negative						1
73	negative						negative						1
74	negative						negative						1
75	negative						negative						1
76	negative						negative						1
77	negative						negative						1
78	negative						negative						1
79	negative						negative						1
80	negative						negative						1
81	negative						negative						1
82	negative						negative						1
83	negative						negative						1
84	negative						negative						1
85	negative						negative						1
86	negative						negative						1
87	negative						negative						1
**Quartet**													
88	negative						negative						1
89	negative						negative						1
90	negative						negative						1
91	negative						negative						1
**Quintet**													
92	negative						negative						1
93	negative						negative						1
94	negative						negative						1
95	negative						negative						1
96	negative						negative						1
